# Surgical Outcomes of 108 Patients Undergoing Holmium Laser Enucleation of the Prostate Over Three Years in a Rural District General Hospital

**DOI:** 10.7759/cureus.99647

**Published:** 2025-12-19

**Authors:** Hari S Bhachoo, Jai S Bhachoo, Viktoria Bajuszova, Arwel T Poacher, Arshad Bhat

**Affiliations:** 1 Surgery, Pinderfields Hospital, Wakefield, GBR; 2 Molecular Biology, School of Molecular and Cellular Biology, University of Leeds, Leeds, GBR; 3 Orthopedics, University Hospital of Wales, Cardiff, GBR; 4 Urology, Hereford County Hospital, Hereford, GBR

**Keywords:** bph, holep, surgery, surgical outcomes, urology

## Abstract

Introduction: An emerging method to treat benign prostatic hyperplasia (BPH) is Holmium Laser Enucleation of the Prostate (HoLEP). This study aims to evaluate the feasibility, safety, and functional outcomes of introducing a HoLEP service in a rural UK District General Hospital (DGH).

Methods: This was a retrospective study of 108 consecutive patients who underwent HoLEP between April 2022 and April 2025, performed by a single surgeon at a rural district general hospital. Perioperative parameters, histology, functional outcomes (International Prostate Symptom Score (I-PSS), quality of life (QoL), maximum urinary flow rate (Qmax), residual volume (RV), and prostate-specific antigen (PSA)), and complications (Clavien-Dindo classification) were analyzed. Nonparametric tests were used to compare preoperative and postoperative data; results are reported with mean differences and P values.

Results: Mean prostate volume was 104 mL, with a mean resected weight of 54 g and a mean operative time of 139 minutes. Significant postoperative improvements were observed across all functional domains: Qmax (+11 mL/s), RV (-196 mL), I-PSS (-12.6 points), QoL (-2.3 points), and PSA (-5.9 ng/mL), all p<0.001. Histology confirmed BPH in 88% and incidental prostate cancer in 7%. Limitations include incomplete follow-up and the absence of a comparator group.

Conclusion: HoLEP can be delivered safely and effectively in a rural DGH, with substantial functional improvements and low rates of clinically significant complications. Broader dissemination of HoLEP beyond tertiary centers is feasible, but comparative and cost-effectiveness studies are required.

## Introduction

Benign prostatic hyperplasia (BPH) refers to the proliferation of prostate stromal and epithelial cells within the transitional zone of the prostate, resulting in compression of the urethra, an increase in prostatic smooth muscle tone, and potential urine outflow obstruction [1]. Consequently, Individuals will suffer numerous urinary tract symptoms. BPH affects approximately 45% of men over 45 years of age, with the prevalence increasing to about 80% in men over 70. Additionally, cases have significantly increased with 112000 global cases in 2021, compared to over 50000 in 1990 [2].

While lifestyle changes and medical treatments can be used to manage symptoms, definitive treatment requires surgical intervention. One such method is Holmium Laser Enucleation of the Prostate (HoLEP) to enucleate prostatic tissue, which is then morcellated and removed from the bladder, widening the urethral channel and improving urinary flow.

The current literature views HoLEP as a less invasive surgical technique compared to open prostatectomy, transurethral resection of the prostate (TURP), and bipolar transurethral enucleation of the prostate (B-TUEP). Reviews of the literature have shown HoLEP to significantly reduce operation time, postoperative recovery, and perioperative blood loss in comparison to its more invasive counterparts. Long-term postoperative outcomes are also satisfactory, with significantly increased urinary flow and a reduction in urinary symptoms. However, there remain ongoing challenges related to the initial setup cost and the learning curve of the procedure, with setup costs exceeding £120000 [3-5].

The aim of this study was to show HoLEP as a viable method of treating BPH using functional outcomes as the primary measure. Additionally, we wanted to show the viability of a HoLEP service in a semi-rural District General Hospital (DGH) setting.

## Materials and methods

All patients who underwent HoLEP between April 2022 and April 2025 at Hereford County Hospital were included in our study. All procedures were performed by the same surgeon. Our study was given ethical approval by Wye Valley NHS Trust Clinical Quality Improvement Department, on which the study was based. Furthermore, all data were anonymized, and participation did not induce any psychological stress or anxiety for patients.

**Figure 1:**
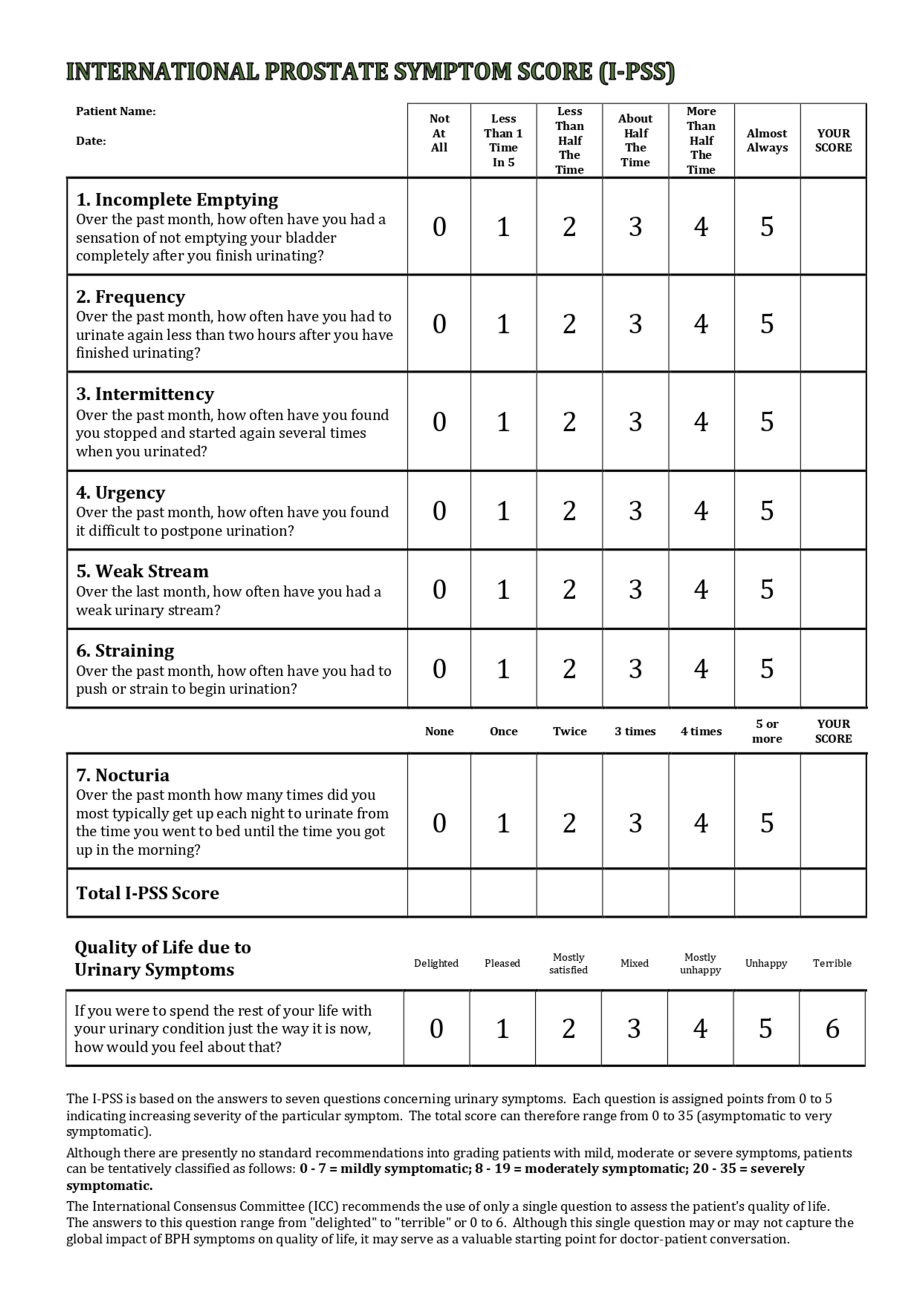
The I-PSS proforma I-PSS: International Prostate Symptom Score Source: [6].

We recorded patient age, indication for surgery, prostate volume with the modality of imaging used, medical treatments patients had tried, and the preoperative prostate-specific antigen (PSA) level, which was compared to postoperative results. Intraoperative measurements included the duration of surgery, volume of prostate removed, and histology findings. We looked at two different quantitative investigations to measure patient outcomes. The first was urodynamic studies, where we compared the preoperative and postoperative residual volume (RV) upon bladder emptying and the maximum rate of urinary flow (Qmax) when passing urine. The second measure used the International Prostate Symptom Score (I-PSS) [6]. This is a globally recognized eight-question proforma given to patients to assess lower urinary tract symptoms associated with BPH (Figure 1). The first seven questions consist of a five-point Likert scale giving a total score out of 35, with higher scores indicating more severe symptoms. The final question, a six-point Likert scale, gauges patients’ quality of life score (QoL) related to their urinary symptoms, with a larger score representing a poorer QoL.

Postoperative outcomes were obtained from outpatient clinic appointments that took place approximately six months postprocedure.

Statistical analysis was performed in GraphPad Prism 10.4.1 (GraphPad Software, Boston, USA) and used to compare preoperative and postoperative patient outcomes, including PSA, I-PSS, QoL, Qmax, and RV values. Data was first assessed to remove outliers using the Rout method. The remaining data was then tested to see if it was normally distributed using the D’Agnostino and Pearson test, the Anderson-Darling Test, the Shapiro-Wilk test, and the Kolmogorov-Smirnov test. Significant differences between mean values were tested using the Wilcoxon Rank.

Patient complications were categorized using the Clavien-Dindo classification. Initially developed in 1992 and updated in 2004, this five-grade scale is widely used to grade postoperative complications in a range of surgical disciplines, including Urology [7]. Grade I complications require no intervention, while grade II complications require therapeutic intervention. Grade III complications require invasive surgical intervention. Grade IV complications are classed as life-threatening and require critical care management. Finally, grade V complications result in patient death.

## Results

Over the three-year period, 108 patients underwent the HoLEP procedure with an average age of 70.7 years (range 50-89 years). Urinary retention was the primary indication for surgery in 37 (34%) of patients, and 90 patients (83%) had already tried medical treatment, one or a combination of finasteride, tamsulosin, mirabegron, and/or oxybutynin. The most common imaging modality used to assess prostate volume was transrectal ultrasound (61 patients), with a mean volume of 104.4 cc. This was followed by MRI (31 patients; mean volume, 104.0 cc) and CT (three patients; mean volume, 116.7 cc).

A total of 110 procedures were performed over the three-year period, with a mean operative time of 139 minutes. The first 55 procedures had a mean operative time of 145 minutes, compared with 133 minutes for the subsequent 55 procedures. Four patients had previous biopsies and had an average HoLEP procedure time of 158.5 minutes. Two patients required reoperation within one week of their first procedure. In the first case, this was due to a malfunction with a morcellation machine. The second case was due to a large prostate, which required additional morcellation. This was the largest amount in the cohort of patients with a weight of 237g. The average weight of prostate removed per patient was 54.3g.

Following histological analysis, 95 patients (88%) were found to have samples consistent with BPH. Eight patients (7%) were found to have evidence of cancer, either Gleason score 3+3 or 3+4. Five patients (5%) were found to have samples with evidence of cell infarction, necrosis, or inflammation.

Patients were scheduled for postoperative follow-up, including urodynamic parameters, I-PSS scores, and PSA testing at six months. Comparable pre- and postoperative data were available for 42 patients for Qmax, 45 for post-void RV, 32 for I-PSS, 35 for QoL, and 50 for PSA.

**Figure 2:**
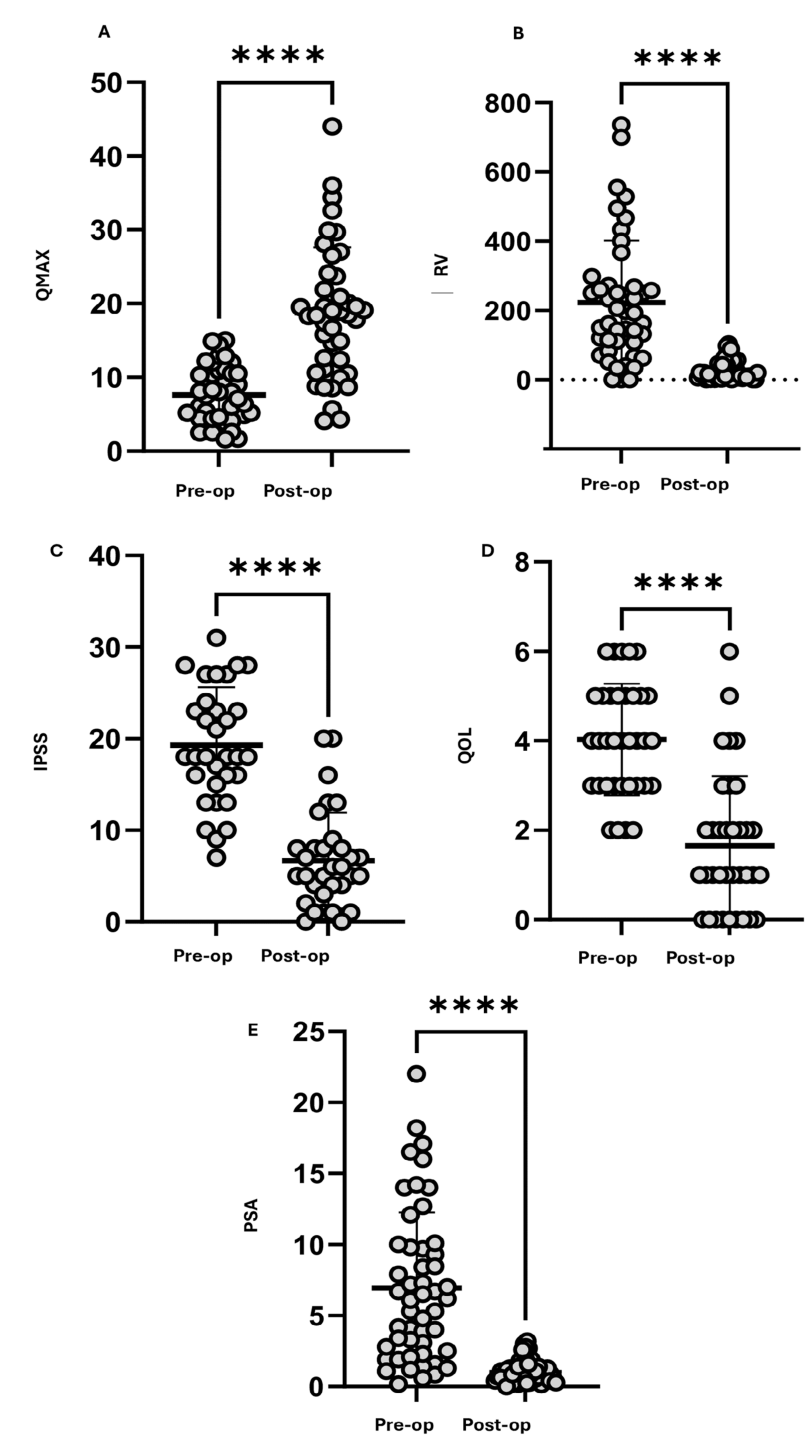
Preoperative and postoperative changes in Qmax, RV, I-PSS, QoL score, and PSA level ^****^p<0.0001. a) Qmax significantly increased from 7.6 mL/s to 18.6 mL/s after surgery. b) RV decreased from 223 mL to 27.4 mL of urine in the bladder post-void. c) There was a significant decrease in patient-reported symptoms, with the I-PSS score decreasing from 19.3 to 6.7 out of 35. d) The QoL score decreased from 4 to 1.7, showing a significant improvement in patient life after HoLEP surgery. e) The postoperative PSA score significantly decreased from 6.9 to 1. Qmax: rate of urinary flow; RV: residual volume; I-PSS: International Prostate Symptoms Score; QoL: quality of life; PSA: prostate-specific antigen Image credit: Author

The average preoperative Qmax score was 7.6 mL/s, which increased to 18.6 mL/s postoperatively (Figure 2A). The average RV significantly decreased from 223.0 mL to 27.4 mL (Figure 2B). Patients also reported fewer urinary symptoms overall following their HoLEP procedure, with a significant decrease in average I-PSS score from 19.3/35 to 6.7/35 (Figure 2C). The average QoL score also significantly decreased from 4.0/6 to 1.7/6 (Figure 2D). Finally, the average PSA value decreased from 6.9 preoperatively to 1.0 postoperatively (Figure 2E). Of note, postoperative values were significantly different from preoperative values across all five measured domains with p<0.0001.

A comparison of the average postoperative RV between the first and second halves of patients showed a decrease from 33 mL to 28 mL. The average I-PSS score also decreased from 9.3 to 8.1, and PSA from 12.3 to 1.3. Qmax saw a decrease between the first and second halves of patients from 21.5 mL/s to 17.6 mL/s. QoL score also slightly increased from 1.6 to 1.7.

Seven patients (6%) developed postoperative complications, evaluated using the Clavien-Dindo classification. Two patients developed incontinence managed conservatively (grade I). One patient required a blood transfusion (grade II) prior to discharge due to a significant postoperative hemoglobin decrease. Three patients developed a urethral stricture. Two patients were treated with dilation, and one required a urethrotomy (grade IIIa/b). Finally, one patient required a return to theatre during their inpatient stay due to the development of hematuria, which was treated with diathermy before discharge (grade IIIb). There were no grade IV or V complications or mortality from any HoLEP procedures.

## Discussion

In conclusion, we have shown HoLEP to be a viable method to treat BPH, with significantly improved patient outcomes, both objective and subjective. Additionally, the procedure resulted in a few complications and zero mortality from six months to two years postoperatively. There remains a gap in the literature for UK-based studies, particularly those conducted in rural District General Hospitals (DGHs), as well as studies that minimise confounding variables, such as ensuring that all procedures are performed by the same surgeon. Our study not only addresses this gap in the literature but also demonstrates similarly positive outcomes associated with HoLEP and its postoperative results when compared with the existing leading literature.

Our study has demonstrated reproducible postoperative outcomes when compared to the leading literature currently available. For example, a large review conducted by Sun et al. found significant improvements in Qmax, RV, QoL, I-PSS, and PSA values following HoLEP surgery [3]. Shvero et al. also found improved functional outcomes with a sustained improvement up to 10 years postoperation from the currently available literature [8]. They also found it to be increasingly common for patients to be discharged on the day of their HoLEP procedure.

Yilmaz et al. concluded that HoLEP was an effective treatment for large prostate sizes ranging from 175 cc to over 300 cc. Once again, they found significant improvements in I-PSS, Qmax, QoL, and RV following HoLEP, which were sustained at up to 18 months postoperation. Retreatment rate remained low at 0-1.3% with only Clavien-Dindo grade I or II complications. In our study cohort, individuals with prostate sizes >175 mL showed similar postoperative improvements in their functional outcomes with no mortality [9].

Further research could compare operative outcomes between different procedures. For example, a comparative study between HoLEP and the current gold standard, TURP, could identify the advantages and disadvantages of each procedure and determine whether specific preoperative variables make patients more suitable for one technique over another. A relatively new procedure, there has been little research in the last few years that has explored this comparison. Of the literature available, HoLEP was shown to have superior postoperative Qmax scores and a greater reduction in postoperative PSA [10]. A study by Fuschi et al. found HoLEP to be superior in prostate volume removed, hemoglobin loss, and recovery time. There was, however, no significant difference in I-PSS and QoL scores [11]. This was also corroborated by Aizezi et al. in men aged over 80 with QoL and I-PSS becoming statistically different in favor of HoLEP at six months postop [12]. In contrast to this, it was found that HoLEP procedures resulted in a higher rate of postoperative complications, such as dysuria and urinary incontinence [13].

The primary limitation of our study was that some patient data remained incomplete, largely relating to postoperative urodynamic studies, I-PSS scores, and follow-up PSA measurements. This was primarily due to many patients not having reached the six-month follow-up threshold and therefore not yet having attended their clinic appointments. Additionally, some patients did not attend their scheduled follow-up appointments. However, with our remaining data, we were still able to show statistical significance. Surgeon experience is also a key part in determining patient outcomes, and as surgical experience increases, adverse outcomes may further diminish.

All procedures in our study were conducted by one surgeon, which may reduce the validity of our results, as it is difficult to interpret the learning curve of HoLEP. However, our results show an improvement in the majority of functional outcomes in the second half of patients undergoing the procedure, suggesting that other surgeons would be able to overcome the learning curve. Nevertheless, future studies should account for surgeon experience and the learning curve of the procedure in the evaluation of patient outcomes and complications.

Finally, our study may restrict external validity given its single-center design; however, we have achieved our aim of showing the ability to set up a functioning HoLEP service at a small DGH. Future studies should aim to be multicenter, in order to achieve a more valid conclusion regarding HoLEP outcomes across DGHs.

## Conclusions

In summary, our study demonstrates HoLEP as a viable operative treatment for BPH among other leading treatments, particularly as we seek new surgical interventions with fewer adverse patient outcomes and more efficient use of operative time. HoLEP has been shown to improve patient symptoms and, by extension, their QoL. Finally, HoLEP is a ubiquitous method for treating BPH, as evidenced by its effectiveness in a rural DGH. This is despite the limitations of our study, which we have acknowledged and for which we have outlined how future studies may address them to provide further validity to our research.

Future studies should look to compare HoLEP with other widely accepted treatment methods for BPH, such as TURP. Furthermore, consideration of setup costs, maintenance of equipment, and the surgeon's learning curve should be accounted for, particularly when providing these services in a DGH.
